# Ecological risk assessment of trace elements accumulated in stormwater ponds within industrial areas

**DOI:** 10.1007/s11356-021-18102-0

**Published:** 2021-12-21

**Authors:** Sylvia Waara, Frida Johansson

**Affiliations:** 1 Department of Environmental and Biosciences, Rydberg Laboratory of Applied Sciences, Halmstad University, Box 823, 301 18 Halmstad, Sweden; 2 Present Address: SWECO Sverige AB, Halmstad, 302 20 Halmstad, Sweden

**Keywords:** Metals, Metalloids, Detention ponds, Sediment quality, Geoaccumulation index, Risk quotients

## Abstract

Stormwater ponds can provide flood protection and efficiently treat stormwater using sedimentation. As the ponds also host aquatic biota and attract wildlife, there is a growing concern that the sediment bound pollutants negatively affect aquatic organisms and the surrounding ecosystem. In this study, we used three methods to assess the accumulation and the potential ecological risk of 13 different heavy metals and metalloids (e.g. trace elements) including both elements that are frequently monitored and some which are rarely monitored in sediment from 5 stormwater ponds located within catchments with predominately industrial activities. Ecological risk for organisms in the older ponds was observed for both commonly (e.g. Cd, Cu, Zn) and seldom (e.g. Ag, Sb) monitored trace elements. The 3 methods ranked the degree of contamination similarly. We show that methods usually used for sediment quality assessment in aquatic ecosystems can also be used for screening the potential risk of other trace elements in stormwater ponds and may consequently be useful in stormwater monitoring and management. Our study also highlights the importance of establishing background conditions when conducting ecological risk assessment of sediment in stormwater ponds.

**Supplementary Information:**

The online version contains supplementary material available at 10.1007/s11356-021-18102-0.

## Introduction

Urban stormwater runoff may cause flooding and pollution in downstream watercourses if neither the flow is reduced nor the pollutants are removed (Baekken 1994; Maltby et al 1995; Blecken et al. 2012; Sharley et al. 2016). Today, stormwater is often retained and treated in wetlands or stormwater ponds (also called stormwater detention ponds or urban ponds), either alone or in combination with other techniques (Marsalek et al. 2005; Blecken et al. 2017; Jefferson et al. 2017; Sharley et al. 2017; Crane 2019). The type and quantity of pollutants in stormwater runoff which accumulates in a specific wetland or stormwater pond depend on the catchment area, climatic factors, land use and percentage impervious surfaces (Färm and Waara 2005; Casey et al. 2006; Frost et al. 2015; Søberg et al. 2016; Blecken et al. 2017; Sharley et al. 2017; Crane 2019).

Recently, concern has been raised over the ecological risks to aquatic life in wetlands and stormwater ponds and for the surrounding wildlife caused by the accumulated sediment. For example, Sharley et al. (2017) sampled 98 wetlands in the Melbourne region and found that catchments with > 10% industrial land use were at a greater risk of containing contaminants at values which exceeded ecological guideline values and waste disposal guidelines. Additionally, a study in Minnesota by Crane (2019) found that industrial areas in Minnesota were more contaminating than residential and commercial areas, leading to statistically significant increases in zinc and some organic micropollutant content in the studied stormwater ponds. This suggests that stormwater ponds in catchments with industrial activity may accumulate high levels of trace elements which could pose a significant risk to the ecosystem.

In studies of trace element accumulation in stormwater ponds, only a few selected heavy metals (e.g. Cd, Cu, Ni, Pb, Zn) are typically monitored (Färm and Waara 2005; Casey et al. 2006; Egemose et al. 2015; Blecken et al. 2017; Sun et al. 2019). However, there are many other potentially ecologically harmful trace element contaminants. Herein, 13 different trace elements representing both commonly and seldom monitored trace elements were selected, and their accumulation in five ponds in catchments with industrial activity was studied. In addition, P and S were analysed to determine whether their concentrations correlated with those of the trace elements, and therefore if they could provide overall contamination values. The patterns and relationships between trace elements with P, S and sediment characteristics were analysed using Principal Component Analysis and Classification. Furthermore, three methods which are commonly used for assessing sediment quality in lakes and watercourses but rarely used for stormwater ponds and wetlands were used to assess the degree of contamination and ecological risk. The accumulation of elements in the ponds compared to background levels was assessed using Müller’s geoaccumulation index, *I*_geo_*,* (Müller 1969), while ecological risk was assessed using Håkanson’s Potential Ecological Risk Index (RI) (Håkanson 1980) and by the Risk Quotient Methodology (RQ-method) (European Chemicals Bureau 2003) which nowadays, under REACH, is called Risk Characterisation Ratios (RCRs) (ECHA 2016) and therefore hereafter referred to as the RCR-method. To our knowledge, *I*_geo_ and RI have not been used to assess risk in stormwater pond sediment. The RCR-method is partly used as sediment guidelines, which in this study serve as PNEC values for calculating RCRs, that are frequently compared to measured values when assessing the quality of stormwater sediment (Färm et al. 2003; Anderson et al. 2004; Färm and Waara 2005; Jang et al. 2010; Blecken et al. 2012; Allen et al. 2017; Sharley et al. 2017; Crane 2019). However, RCRs have not been calculated nor evaluated in previous studies. In summary, in this study, we use three methods to assess the accumulation and the potential ecological risk of 13 different heavy metals and metalloids (e.g. trace elements) including both elements that are frequently monitored and some which are rarely monitored in sediment from 5 stormwater ponds located within catchments with predominately industrial activities. By combining the results from each method, the ponds at the highest risk and the trace elements of highest concern were identified and provided essential guidance for our continued contaminant monitoring.

## Materials and methods

### Study site

**Fig. 1:**
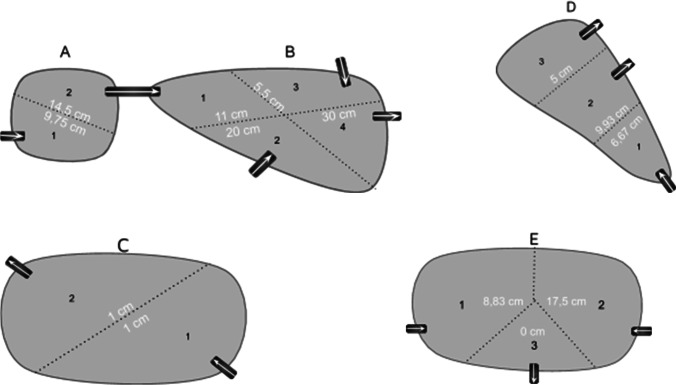
Design of the ponds and the measured thickness of sediment

The sampled stormwater ponds are in the municipality of Ängelholm in southern Sweden. The characteristics of the ponds and their catchment are presented in Table 1, and their design and sediment thicknesses are presented in Fig. 1. The ponds were designated Pond A and B (DUM 20 and DUM3, Åkerslund 26:3), pond C (DUM 23, Rebbelberga 14:6), pond D (DUM 8, Rebbelberga 19:2) and pond E (DUM 19, Munka-Ljungby 52:20), and they are located with catchments with mainly industrial activity. The industrial areas have been developed at different paces from when the respective ponds were built, and consequently the percentage impervious area has been changing over time. The industrial activity is diverse, and it consists of workshops, shops and storage of building materials including metals, storage of vehicles for building and construction work, haulage companies, a bus company, car testing facilities, the major recycling plant in Ängelholm and the power plants of Ängelholm and Munka Ljungby. Pond A–D are located less than 1 km apart, and pond E is located about 5 km east of the other ponds in the village of Munka Ljungby. The stormwater ponds were designed to prevent flooding in downstream areas.

**Table 1:** Characteristics of the investigated ponds. Data obtained from the Municipality of Ängelholm

Pond	Area (m^2^)^1^	Catchment area (ha)^2^	% Impervious surface^1^	The ratio (in percentage) of pond area to impervious area	Age 2019 (years)
A (DUM 20)	300	6.5	50	0.9	6
B (DUM 3)	950	72.0	50	0.3	17
C (DUM 23)	2000	14.5	12^1^	1.1	2
D (DUM 8)	1300	44.3	30	1.0	18
E (DUM 19)	500	24.8	70^2^	0.3	2

^1^The impervious surface will increase when the area is fully developed

^2^The pond was constructed after the development of the industrial area

### Sediment sampling and analysis

Sediments were first sampled in April 2019. Each pond was split into as many sections as there were inlets and outlets (Fig. 1), which enabled the identification of differences in sediment characteristics and pollutant levels within a single pond. Sediment depth and sample collection were conducted using the method described by Blecken et al. (2017). Briefly, sediment samples were taken with a sediment retriever. The depth of the accumulated sediment layer (i.e. only that which was above the clay layer) was measured with a ruler while the extracted core was still in the retriever. After the sediment sample was emptied into a clean bucket, the clay layer was carefully removed, and the remaining sample was mixed thoroughly with the other 3–6 subsamples collected from the same pond section. The number of subsamples taken from each section was dependent on the section area.

In August 2019, surface soil around the ponds A + B, C and E and well above possible waterlines was collected to determine background element concentrations in soil which in this area is dominated by kaolinite clay mineral. Subsurface soil was collected randomly using a small, clean, plastic spoon and then mixed thoroughly before analysis. At this time, a sample from E3 was also taken as it was not possible to retrieve a sample with the core sampler during the first sampling because this part of the pond is covered with macadam. Additionally, although no accumulation of sediment was evident above the macadam, it was possible to collect the material deposited around and under the macadam layer and around the macrophyte roots using a gloved hand in order to avoid sampling the underlying clay layer.

The particle size distribution of the samples was determined according to ISO 11277:2009 “Soil quality—Determination of particle size distribution in mineral soil material—Method by sieving and sedimentation”. The determination of dry matter was conducted according to Swedish Standard (SS) 28,113 “Determination of dry matter and ignition residue in water, sludge and sediment”. The elemental composition of the samples was analysed with inductively coupled plasma sector field mass spectroscopy (ICP-SFMS). In all cases, samples were digested in concentrated acid. For the elements As, Ba, Cd, Co, Cr, Cu, Hg, Ni, P, Pb, S, V and Zn, samples were heated and mixed in concentrated HNO_3_, whereas for Ag and Sb, samples were treated with *aqua regia* (3:1 HCl:HNO_3_). General guidelines of the method are found in SS EN ISO 17294–1 “Water quality—Application of inductively coupled plasma mass spectrometry (ICP-MS)—Part 1: General guidelines.” Analysis was conducted according to ISO 17294–2:2016 “Water quality—Application of inductively coupled plasma mass spectrometry (ICP-MS)—Part 2: Determination of selected elements including uranium isotopes (modified)” and USA EPA 200.8 “Determination of Trace Elements in Waters and Wastes by Inductively Coupled Plasma-Mass Spectrometry”. All analyses were carried out by a certified laboratory.

The division of trace elements into commonly monitored (i.e. Cd, Cr, Cu, Hg, Ni, Pb, Zn) and seldom monitored (Ag, As, Ba, Co, Sb, V) was related to the frequency the elements were quantified in literature studies such as those presented in Table 5.

### Data analysis

Calculations and statistical analyses were conducted using Statistica 13.5.0.17, TIBCO Software Inc. Multivariate analysis was conducted using the module Principal Component Analysis and Classification. Computational details are described in the software in the document ¨Principal Components & Classification Analysis – Computational Details¨.

### Determination of the contamination degree using the geoaccumulation index (_*Igeo*_)

The single element geoaccumulation index*, I*_geo_, developed by Müller (1969) is generally used for comparing current metal levels in sediment to pre-industrial levels (Zhu et al. 2013; Manoj and Padhy 2014; Duodo et al. 2016; Wang et al. 2016, 2018; Li et al. 2020). It is calculated using Eq. 1:

1 $${I}_{\mathrm{geo}}={\mathrm{log}}_{2}\left(\frac{{C}_{\mathrm{i}}}{1.5 x {B}_{\mathrm{i}}}\right)$$

where *c*_i_ is the measured concentration of the examined metal in the sediment, and *B*_i_ is the geochemical background concentration. Here, *B*_i_ was calculated as the average value obtained from three soil samples taken around ponds A + B, C and E. An adjustment factor of 1.5 was used to account for possible variations in background values as well as minor anthropogenic influences. Müller (1969) used a graded scale of classification (Table 2).

**Table 2:** Classification of *I*_*geo*_ (Müller 1969)

Class	Description	*I*_geo_ value
0	Unpolluted	*I*_geo_ ≤ 0
1	Slightly polluted	0 < *I*_geo_ ≤ 1
2	Moderately polluted	1 < *I*_geo_ ≤ 2
3	Moderately/severely polluted	2 < *I*_geo_ ≤ 3
4	Severely polluted	3 < *I*_geo_ ≤ 4
5	Severely/extremely polluted	4 < *I*_geo_ ≤ 5
6	Extremely polluted	*I*_geo_ > 5

The sum of the average *I*_geo_ values for all trace elements determined in each pond were calculated and used to generate a risk ranking for each respective pond. These values were then compared to values obtained from two other risk ranking methods described below. Negative values (i.e. unpolluted; class 0) were set to zero.

### Ecological risk assessment using the potential ecological risk index (RI)

The RI method was developed by Håkanson (1980) for assessing the potential ecological risks in sediment in freshwater ecosystems in Sweden. This was determined from the quantity of 7 elements (As, Cd, Cr, Cu, Hg, Pb and Zn) and one persistent organic pollutant (PCB), and continues to be used for ecological risk assessments of heavy metals in sediments (Manoj and Padhy 2014; Duodu et al. 2016; Jiao et al. 2017; Wang et al. 2018; Wei et al. 2019; Li et al. 2020). The RI method considers the hazard of metals to humans and ecosystems from two aspects—the abundance principle and the release effect—where the potential toxicity of a metal is inversely proportional to its abundance. The index includes the risk factor, ${Er}^{i}$, for a given substance (Eq. 2), the sum of which being the Potential Ecological Risk Index (RI) (Eq. 4; Håkanson 1980):

2 $${Er}^{i}= {Tr}^{i}\times {C}_{f}^{i}$$

3 $${C}_{f}^{i}= \frac{{C}_{o}^{i}}{{C}_{n}^{i}}$$

4 $$RI= \sum\nolimits_{i}^{n}{E}_{r}^{i}= \sum\nolimits_{i}^{n}{T}_{r }^{i} \times {C}_{f}^{i}$$

where ${Tr}^{i}$ is the toxic response factor of substance *i,* ${C}_{f}^{i}$ =is the contamination factor of substance *i,* ${C}_{o}^{i}$ is the concentration of substance *i* in the sediment and ${C}_{n}^{i}$ = concentration of substance *i* in the background.

Here, ${C}_{f}^{i}$ was calculated as the average value obtained from 3 soil samples taken around ponds A + B, C and E. The toxic response factors (*Tr*) for each element were obtained from previously published studies (Table 3). For a single substance, the risk factor value (*Er*) and the sum of risk factors (RI) were classified according to Håkanson (1980), Table 4.

**Table 3:** Toxic response factors used for calculating the Potential Risk Index (Håkanson 1980)

Element	*Tr*	Source
Ag	17.5	Aksu et al. 1998
As	10	Håkanson 1980
Ba	2	Yang et al. 2015
Cd	30	Håkanson 1980
Co	5	Zhang et al. 2017
Cr	2	Håkanson 1980
Cu	5	Håkanson 1980
Ni	5	Zhang et al. 2017
Pb	5	Håkanson 1980
Sb	7	Wang et al. 2018
V	2	Zhu et al. 2013
Zn	1	Håkanson 1980

**Table 4:** Classification of Potential Ecological Risk Index (Håkanson 1980)

Level of potential ecological risk	${Er}^{i}$ value	Level of ecological risk	$\mathrm{RI}$ value
Low	${Er}^{i}$< 40	Low	RI < 150
Moderate	40 ≤ ${Er}^{i}$< 80	Moderate	150 ≤ RI < 300
Considerable	80 ≤ ${Er}^{i}$< 160	Considerable	300 ≤ RI < 600
High	160 ≤ ${Er}^{i}$< 320		
Very high	${Er}^{i}$≥ 320	Very high	RI ≥ 600

### Ecological risk assessment using the risk characterisation ratio method (RCR-method)

The derivation of RCRs (the term Risk Quotients is also commonly used) for assessing ecological risk is used in many fields, such as for evaluating the risk of chemicals within the EU (ECHA 2016), the risk of emerging pollutants in landfill leachate (Nika et al. 2020) or the potential risk of pharmaceuticals in surface waters (Zhou et al. 2019). The RCR is derived by dividing the Predicted or Measured Environmental Concentration (PEC or MEC, respectively) by the Predicted No Effect Concentration (PNEC), where a RCR ≥ 1 indicates a risk of deleterious effects to the ecosystem by the pollutant in question. The PNEC is usually derived using toxicity data for sediment dwelling organisms, but where this data is lacking other methods such as the Equilibrium Partitioning Model which use toxicity data for pelagic species can be used (CIS 2011). PNEC values are often generated when Environmental Quality Standards are set, such as those under the Environmental Quality Standard Directive of the EU (Directive 2013/39/EU). In this study, we use data from when EQS were set for metals and metalloids in water, soil and sediment in the Netherlands (Crommentuijn et al. 2000), as equivalent data are still lacking for most sediment contaminants in Swedish and European legislation. Models and data from the Netherlands have also been used for developing guideline values for contaminated soil in Sweden (Swedish Environmental Protection Agency 2009). An RCR was consequently calculated using Eq. 5:

5 $${RCR}_{i}=\frac{{MEC}_{i}}{{NC}_{i}}$$

where *RCR*_*i*_ is the risk characterisation ratio for element *i, MEC*_*i*_ is the measured concentration of element *i* in the sample and *NC*_*i*_ is the negligible concentration of element *i.* Values for NC_i_ were calculated using the modified Eq-P method and take into account mixture effects. Elements at concentrations lower than or equal to their *NC* is not expected to cause negative long-term effects in the ecosystem (Crommentuijn et al. 2000). NCs were available for all trace elements except Ag, for which the Danish Default Guideline Value (DGV) of 1.5 mg per kg of dry weight (mg/kg DW) for sediments was used as PNEC value (Ministry of Environment and Food of Denmark. 2017).

The sum of average RCR values for all trace elements in each pond were calculated to enable risk ranking with *I*_geo_ and RI.

### Remediation requirements

The samples were also classified using the generic guideline values for contaminated soil developed by the Swedish Environmental Protection Agency (2009) using the guideline values in the updated list from 2016 (Swedish Environmental Protection Agency 2016). Generic guideline values were available for all trace elements except Ag (Supplementary Data Table S1). Two different generic guideline values are derived depending upon expected land use, sensitive land use (KM) and less sensitive land use (MKM). KM values are used for example if the land is going to be used for housing, while MKM values are used if the land use is intended for industries.

## Results and discussion

### Concentrations of elements in sediment

**Fig. 2:**
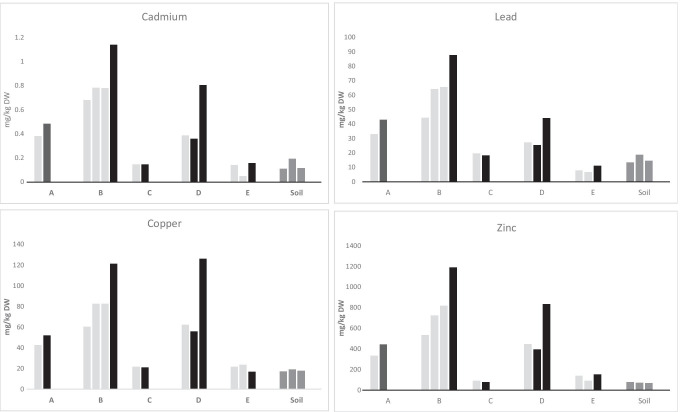
Concentrations of four commonly monitored trace elements (Cd, Co, Pb, Zn) in sediments in stormwater ponds and soil from the banks of the ponds in areas with industrial activity. Light grey bars—inlets, black bars—outlets

**Fig. 3:**
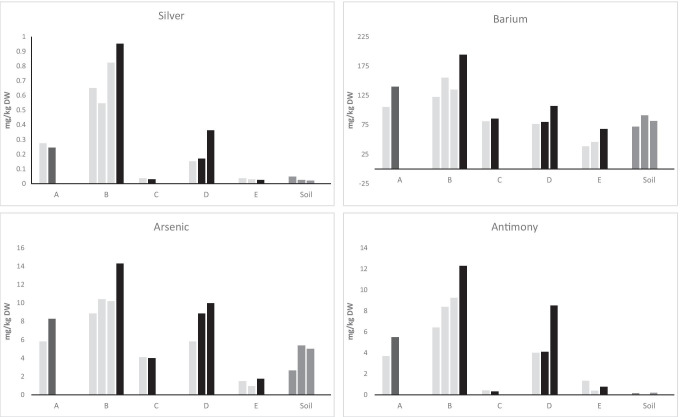
Concentrations of four seldom monitored trace elements (Ag, As, Ba, Sb) in sediment in stormwater ponds and soil from the banks of the ponds in areas with industrial activity. Light grey bars—inlets, black bars—outlets

The concentrations for elements at all sampling sites and the average values for each element in each pond are presented in Supplementary Data Table S2. The measured concentrations of four commonly monitored (Cd, Cu, Pb and Zn) and four seldom measured (Ag, As, Ba and Sb) trace elements in stormwater ponds are presented in Figs. 2 and 3, respectively. For all elements, the highest concentrations were recorded in the older ponds (A, B and D), while the concentrations in recently constructed ponds (C and E) were generally low or close to the concentration found in the surrounding soil. The mean element concentrations were ranked as follows: P > S > Zn > Ba > Cu > V > Pb > Cr > Ni > Co > As > Sb > Cd > Ag. Measured concentrations for Hg never exceeded the detection limit (0.5 mg/kg DW), while Ag was below the detection limit (0.7 mg/kg DW) in sediments from pond C and E.

The element concentrations were generally similar or higher at the outlets compared to the inlets of ponds. For pond A, samples from near the outlet had higher concentrations for all elements compared to the inlet except for S. In pond B, all elements were found in higher concentrations near the outlet, except for Co, Cr and V which were in a higher concentration around inlet B2. In pond D, all elements were found in higher concentrations at outlet D3 compared to the inlet, but only Cd, Cu, Pb, S and Zn were higher in concentration at outlet D2 compared to the inlet. In pond E, element concentrations, except for Ag, Co, Cu, S and Sb, were generally higher at the outlet than at the inlets. Inlet E2 presented the highest concentration of Co and Cu, while inlet E1 had the highest concentrations of Ag, S and Sb. Finally, in pond C, a different trend was observed where Ag was below the detection limit and all other element concentrations—except for those of Ba, Cr and V—were higher at the inlet than the outlet.

Our measured trace element concentrations were then compared with data from other studies (Table 5). The concentrations of commonly monitored trace elements (i.e. Cd, Cr, Cu, Ni, Pb, Zn) were detected within the same order of magnitude as those observed in several previous international and national studies (Jang et al. 2010; Blecken et al. 2012, 2017; Istenič et al. 2012; Egemose et al. 2015; Frost et al. 2015; Sharley et al. 2017; Crane 2019; Sun et al. 2019), but lower maximum concentration values were obtained here compared to those found in ponds within industrial areas (Sharley et al. 2017). Blecken et al. (2012) also detected sixfold higher Cr concentrations in sediment close to stormwater outlets, while Jang et al. (2010) observed fourfold higher maximum values in road residues. The concentration values for seldom monitored metals (i.e. Ag, As, Ba, Co, Sb, V) were also within the same order of magnitude as previously reported values (Färm et al. 2003; Jang et al. 2010; Frost et al. 2015; Sharley et al. 2017; Crane 2019). Furthermore, higher maximum concentration values for these elements were found in stormwater ponds and tunnels within industrial areas (Färm et al. 2003; Sharley et al. 2017). Jang et al. (2010) generally observed lower maximum values in sediment from stormwater ponds, but the maximum concentrations of Ba was higher.

**Table 5:** Concentrations of trace elements in sediments from stormwater ponds, outlets and stormwater tunnels. Data is for stormwater ponds if not stated. Min–max values are presented or if not available mean ± standard deviation is used

Referen-ce	Ag	As	Ba	Cd	Co	Cr	Cu	Hg	Ni	Pb	Sb	V	Zn
This study *N* = 5	0.030–0.952	0.9–14.3	39–194	0.05–0.80	3.5–13.2	9.0–45.6	20.9–126.0	< 0.5	6.4–34.9	6.5–87.6	0.325–12.3	15.9–75.3	78.6–1190
Crane 2019 *N* = 15	0.99 ± 2.4	5.5 ± 2.3	147 ± 43	1.5 ± 0.57	8.2 ± 2.8	42.0 ± 14.7	31.6 ± 10.1	0.059 ± 0.022	23.3 ± 7.2	39.2 ± 25.5	< 1.2	45.3 ± 11.7	154.3 ± 79.7
Sun et al. 2019 *N* = 12	n.a	n.a	n.a	n.a	n.a	15–86	18–200	n.a	17–110	9.9–76	n.a	n.a	57–850
Blecken et al. 2017 *N* = 25	n.a	n.a	n.a	< 0.1–2.3	n.a	2–81	3–109	n.a	2–39	3–77	n.a	n.a	14–597
Sharley et al. 2017 *N* = 98	< 2–6	< 5–51	40–430	< 1–11	2–31	12–121	6–1090	< 0.1–2.9	4–159	9–456	< 5–17	14–130	12–4940
Ege–mose et al. 2015 *N* = 37	n.a	n.a	n.a	0.1–1.4	n.a	18–62	18–62	n.a	18–62	18–62	n.a	n.a	166–451
Frost et al. 2015	0.04–0.37	n.a	64.7–507.3	0.06–0.39	3.8–20.3	9.6–60.5	9.5–42.3	n.a	6.6–45.4	6.0–38.1	n.a	14.3–67.5	35.5–175.4
Blecken et al. 2012 *N* = 32	n.a	n.a	n.a	0.15–1.23	3.8–25.0	64–307	17–263	n.a	10–50	8–42	n.a	n.a	68–470
Istenič et al. 2012 *N* = 3	n.a	n.a	n.a	< 0.5	n.a	17–80	4–3293	n.a	10–42	< 2–220	n.a	n.a	26–1361
Jang et al. 2010 *N* = 22	< 0.8	0.6–24.8	8.1–1019	< 0.37 1st–5.3	n.a	5.8–174.5	4.5–90.4	< 0.02	5.4–40.4	5.6–196	n.a	n.a	5.4–711
Färm et al. 2003 *N* = 3	0.314–1.70	10.2–20.7	n.a	1.15–2.93	19.6–34.7	51.0–75.7	106–159	Kan finnas	32.0–50.5	61.4–98.2	n.a	73.2–106	609–974

*n.a.* not analysed, *N* the number of ponds studied

^1^Wetlands

^2^Stormwater outlets

^3^Road residues from stormwater systems

^4^Sediment from stormwater tunnels

### Sediment depth and particle size distribution

The sediment depth measurements in different ponds showed that there were no significant differences in sediment depth between the areas surrounding the inlets and outlets (Fig. 1). The largest sediment depths were observed in one of the older ponds (B). Accumulation of material had already occurred at the inlets in one of the newer ponds (E), while the accumulation was still low in the other new pond (C).

**Fig. 4:**
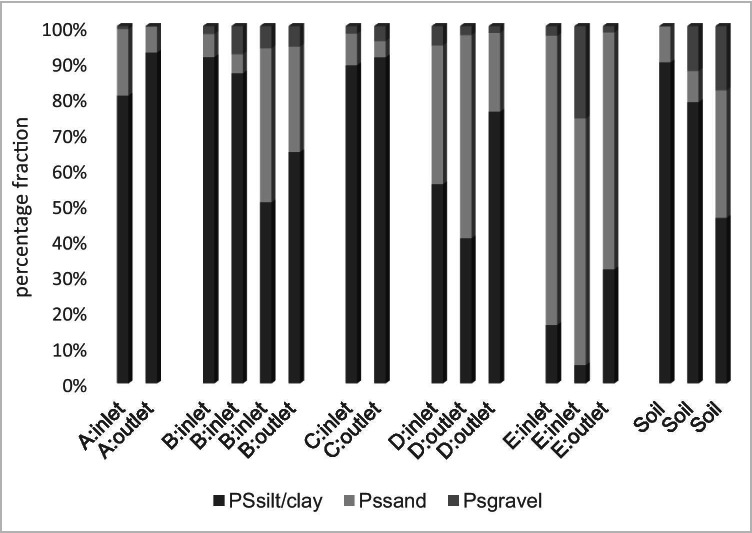
Particle size distribution of the sediment in the different sampling sites in five stormwater ponds. Particle size (PS) for PSsilt/clay < 0.063 mm, PS sand 0.063- 2 mm and PS gravel > 2 mm

The particle size distribution and the sediments varied between sampling sites across individual ponds and between ponds (Fig. 4, all data shown in Supplementary Data Table S2). Most of the sampling sites contained mainly fine material (PSsilt/clay), except for pond E. During sampling in August 2019, erosion of material from the surrounding step banks into the ponds was observed, and thus the sediment may constitute material from both the catchment and the areas directly surrounding the ponds.

A well-designed pond should accumulate coarse material at the inlets and fine material at the outlets, but we observed mixed results. Here, the stormwater ponds were constructed to prevent flooding downstream and have several inlets (e.g. B and E) or several outlets (e.g. D); therefore, the hydraulic efficiency may be low. However, previous studies also reported varied results which suggested that fine sediment may also accumulate preferentially at inlets in ponds with high hydraulic efficiency (Istenič et al. 2012; Blecken et al. 2017).

### Pattern and relationship between elements and sediment characteristics

**Fig. 5:**
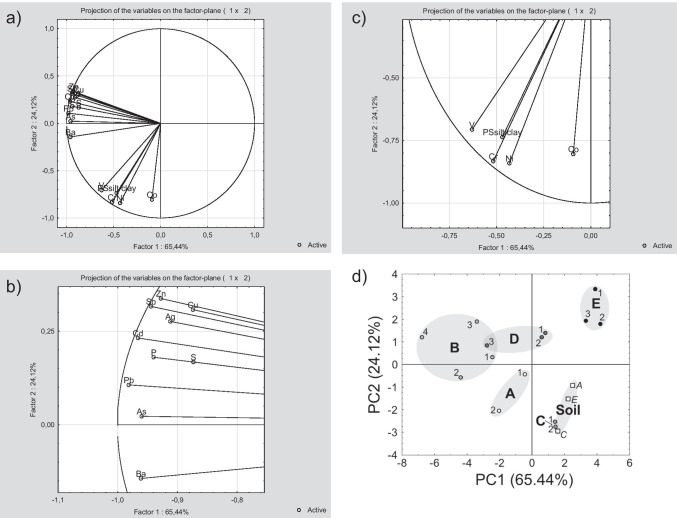
**a**) Projection of the variables on PC 1 and PC2. **b**) Enlarged section of PCA with Group A elements. **c**) Enlarged section of PCA with Group B elements and PSsilt/clay. **d**) Projection of the samples on PC1 and PC2

Trends in variation and the relationship between measured variables were further explored using PCA and classification methods. PCA on all element concentration data and sediment characteristics was not possible because the correlation matrix became singular indicating that the data could be replicated as the linear combination of fewer variables. Therefore, PCA was conducted with the element concentration and PSsilt/clay data (Fig. 5). In Fig. 5a, the PCA for the 2 first Principle Components is presented for the samples including the soil samples (cases *n* = 17), the 12 trace elements detected, P, S and the silt/clay fraction (variables *n* = 15). Only 2 PCs had an eigenvalue above 1, and PC1 and PC2 describe 65.44% and 24.12% of the variation respectively. Two distinct groups of variables were identified (Fig. 5a). The first group (Fig. 5b), hereafter labelled Group A, consisted of elements with a high negative loading (score) on PC1 include (in descending order) Pb, Cd, Ba, As, Sb, P, Zn, Ag, Cu and S. They also all have a low positive loading on PC2 except for Ba. The second group, hereafter labelled B (Fig. 5c), consisted of elements and parameters with lower negative loadings on PC1 and higher negative loadings on PC2 included (in descending order of PC 1) V, Cr, PSsilt/clay (i.e. PS < 63 µm) and Ni. Co was included in the second group, but we remark that it showed a very low negative loading on PC1.

The projection of the cases on PC1 and PC2 showed that samples from a given pond were loosely grouped (Fig. 5d). This suggested that samples taken from a single pond were more similar to each other than to those from other ponds, which reflects differences in catchment characteristics. Samples with the highest contamination level had the highest negative loading in PC1 (i.e. located furthest to the left in Fig. 5b), which indicated that pond B was the most contaminated and pond E was the least contaminated.

A Pearson’s *r* correlation analysis was also conducted to identify correlations between the elements and the sediment characteristics including PSclay/silt (i.e. PS < 63 µm), PSsand (i.e. PS 0.63 µm–2 mm), PSgravel (i.e. PS > 2 mm), PScoarse material (i.e. PSsand and PSgravel) and sediment depth (Table 6). Statistically significant positive correlations (*p* ≤ 0.05) were observed for PSclay/silt and (in order of decreasing correlation) V, Cr Ni, S, Ba and Co, where the same elements also showed significant negative correlation with similar amplitude with PSsand. No significant correlation between PSgravel and the elements was observed. Additionally (in order of decreasing correlation) P, Pb, Ba, Cd, Sb, Zn, Ag and S were significantly correlated with sediment depth. Finally, a correlation analysis was also conducted for the trace elements and the potential indicators of contamination P and S (Table 6). Elements constituting Group A as identified in the PCA (see Fig. 5a,b) showed higher correlation coefficients with P and S than those in Group B (Fig. 5a,c) except for V which showed a significant correlation with S. Group A elements were more strongly correlated with P than with S.

**Table 6:** Pearson *r* correlations between sediment characteristics and elements. Significant correlations are presented in in bold. Statistical significance was set to < 0.05

Element mg/kg DW	PSsilt/clay^1^ (%)	PSsand^2^ (%)	Sediment depth (cm)	P mg/kg DW	S mg/kg DW
Ag	0.29	− 0.31	**0.57**	**0.96**	**0.78**
As	0.48	− 0.47	0.53	**0.90**	**0.82**
Ba	**0.58**	− **0.59**	**0.60**	**0.91**	**0.87**
Cd	0.38	− 0.38	**0.59**	**0.93**	**0.86**
Co	**0.54**	− **0.68**	0.16	0.10	0.15
Cr	**0.91**	− **0.92**	0.16	0.46	**0.59**
Cu	0.28	− 0.28	0.48	**0.76**	**0.75**
Ni	**0.85**	− **0.86**	0.14	0.42	0.47
P	0.38	− 0.40	**0.62**	–––-	**0.84**
Pb	0.44	− 0.44	**0.61**	**0.96**	**0.86**
S	**0.59**	− **0.58**	**0.54**	**0.84**	–––-
Sb	0.31	− 0.31	**0.59**	**0.92**	**0.84**
V	**0.93**	− **0.92**	0.16	0.49	**0.70**
Zn	0.26	− 0.26	**0.58**	**0.89**	**0.80**

^1^Particle size < 0.063 mm

^2^Particle size 0.063 mm–2 mm

### Contamination degree using the geoaccumulation index *I*_*geo*_

**Fig. 6:**
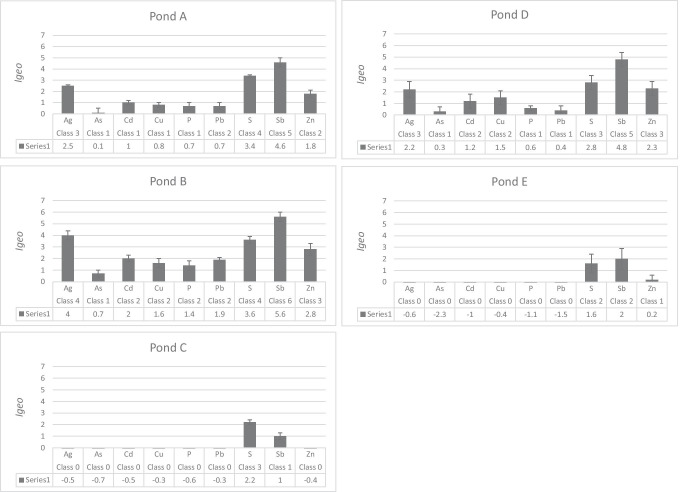
Contamination degree for trace elements in the different ponds using the geoaccumulation index *I*_geo_. Classification was made using mean values, and error bars present standard deviation

The single element index, *I*_geo_ was used to assess the degree of contamination in the sediment. The mean *I*_geo_ values for the elements Co, Cr, Ni and V in all ponds were all negative, and the sediment was therefore classified as unpolluted (Class 0) with respect to these elements. The mean *I*_geo_ for Ba only exceeded 0 in pond B (0.3 ± 0.3). The *I*_*geo*_ for the elements Ag, As, Cd, Cu, P, Pb, S, Sb and Zn are shown for each pond in Fig. 6. The complete data set for *I*_geo_ values is shown in Supplementary Data Table S3. The highest mean *I*_geo_ values were obtained for Ag, S, Sb and Zn. The older ponds (A, B and D) showed higher contamination degree than the newest ponds (C and E). Overall, Pond B was the most polluted, showing the highest *I*_geo_ values for Ag (Class 4), S (Class 4) and Sb (Class 6) and Zn (Class 3). Ponds C and E only showed contamination by S and Sb (both ponds) and Zn (pond E only).

### Ecological risk assessment using the Potential Ecological Risk Index (RI)

The mean risk factor, *Er,* for the elements As, Ba, Co, Cr, Cu, Ni, Pb, V and Zn, was below 40 which suggested they provided low potential ecological risk. The *Er* values for Ag, Cd and Sb are shown in Table 7, and all data are summarised in Supplementary Data Table S4. Low risk factors (*Er* < 40) were obtained for the newer ponds C and E, while the older ponds A,B and D showed very high risks (*Er* ≥ 320) for Ag and Sb.

**Table 7:** Mean risk factor, *Er,* values for Ag, Cd and Sb with standard deviation; the RI for the sum of risk factors and the contribution, in percentage, of Ag, Cd and Sb to the RI in sediment from the five stormwater ponds

Pond	*Er*-Ag^1^	*Er*-Cd^1^	*Er*-Sb^1^	RI^2^	% Ag of RI	% Cd of RI	% Sb of RI	% Ag + Cd + Sb of RI
A	147 ± 11 Class 3	93 ± 16 Class 3	267 ± 75 Class 4	593 Class 3	25	16	45	86
B	421 ± 102 Class 5	182 ± 44 Class 4	528 ± 143 Class 5	1248 Class 4	34	15	42	91
C	18 ± 2 Class 1	31 ± 0 Class 1	22 ± 4 Class 1	134 Class 1	14	23	16	53
D	129 ± 66 Class 3	111 ± 54 Class 3	321 ± 150 Class 5	646 Class 4	20	17	50	86
E	17 ± 3 Class 1	25 ± 12 Class 1	48 ± 27 Class 2	117 Class 1	16	20	39	75

^1^The risk of single element was classified as according to Håkanson (1980), and here, for convenience, it has been given a numeric classification: Class 1 Low potential ecological risk *Er* < 40; Class 2 Moderate potential ecological risk 40 ≤ *Er* < 80; Class 3 Considerable ecological risk 80 ≤ *Er* < 160; Class 4 High potential ecological risk 160 ≤ *Er* < 320 and Class 5 Very high ecological risk *Er* > 320

^2^The RI, the sum of the risk factors, was classified according to Håkanson (1980), and here, for convenience, it has been given a numeric classification: Class 1 Low ecological risk RI < 150; Class 2 Moderate ecological risk 150 ≤ RI < 300; Class 3 Considerable ecological risk 300 ≤ RI < 600; Class 4 Very high ecological risk RI ≥ 600

The RI for ponds C and E were classified as low, while the classifications for RI for ponds A, B and D ranged from considerable ecological risk (Class 3) to very high ecological risk (Class 4). The highest RI was obtained in pond B.

### Ecological risk assessment using the RCR-method

Elements with mean RCRs exceeding 1 in at least one sample are summarised in Table 8, while all data is presented in Supplementary Data Table S5. Mean RCRs in soil were < 1 for all elements except for Co and V. In pond sediment, mean RCRs for Ag, As, Cd, Cr, Pb were always ≤ 1. For Ba, two samples (B2 and B4) had a RCR ≥ 1 (1.0 and 1.2, respectively). For Cd and Ni, only samples B4 and C1 had a RCR ≥ 1 (both 1.0). For Co and V all ponds except pond E had a mean RCRs of 2 > RCR ≥ 1. Finally, mean RCRs for Cu, Sb and Zn exceeded 2 in numerous samples, where the highest mean RCRs for Zn (5.6), Sb (2.8) and Cu (2.4)—and therefore the highest Sum-RCR—were obtained in pond B. The lowest sum-RCR values were obtained for the newest ponds C and E. Indeed, the sum-RCR for the surrounding soil exceeded that of pond E.

**Table 8:** Mean Risk Characterisation Ratio (RCR) with standard deviation for elements measured in soil and sediments in five stormwater ponds. Negligible concentrations (NC, in mg/kg DW sediment) was collected from Crommentuijn et al. (2000). Mean RCRs in bold text ≥ 1, ponds with a at least 1 sample with a RCR ≥ 1 in italics

Pond	Ba (157)^1^	Cd (1.1)	Co (9.1)	Cu (36)	Ni (35)	Sb (3.2)	V (42)	Zn (145)	Sum-RCR^2^
A	*0.8* ± *0.2*	0.4 ± 0.1	**1.2 ± 0.2**	**1.3 ± 0.2**	0.7 ± 0.1	**1.4 ± 0.4**	**1.6 ± 0.3**	**2.7 ± 0.5**	11.1 ± 2.1
B	1.0 ± 0.2	*0.8* ± *0.2*	**1.1 ± 0.2**	**2.4 ± 0.7**	0.7 ± 0.2	**2.8 ± 0.8**	**1.3 ± 0.2**	**5.6 ± 1.9**	17.3 ± 4.2
C	0.5 ± 0.0	0.1 ± 0.0	**1.4 ± 0.0**	0.6 ± 0.0	**1.0 ± 0.0**	0.1 ± 0.0	**1.3 ± 0.1**	0.6 ± 0.1	6.3 ± 0.1
D	0.6 ± 0.1	0.5 ± 0.2	**1.0 ± 0.2**	**2.3 ± 1.1**	0.5 ± 0.1	**1.7 ± 0.8**	**1.0 ± 0.2**	**3.8 ± 1.6**	12.3 ± 4.5
E	0.3 ± 0.1	0.1 ± 0.1	*0.8* ± *0.5*	0.6 ± 0.1	0.3 ± 0.1	0.3 ± 0.1	0.5 ± 0.2	*0.9* ± *0.2*	3.9 ± 0.4
soil	0.5 ± 0.1	0.1 ± 0.0	**1.4 ± 0.3**	0.5 ± 0.0	0.8 ± 0.2	0.0 ± 0.0	**1.1 ± 0.1**	0.5 ± 0.0	5.6 ± 0.8

^1^NC in parenthesis

^2^Sum mean RCR for all elements analysed except P and S

### Remediation requirements

The complete data set is shown in Supplementary Data Table S2. The mean concentration of each element in background soil was below the respective generic guideline values for both less sensitive land use (MKM) and sensitive land use (KM). The KM value for Co was, however, exceeded in the surrounding soil from pond C. The generic guideline value for less sensitive land use (MKM) was never exceeded in pond sediments except for Zn where all samples from pond B exceeded the MKM value as did the sample D3 from pond D. Based upon means, the KM value was exceeded in pond A for Zn, for pond B for As, Cd, Cu, Pb and Zn (i.e. exceeds MKM) and for pond D for Cu and Zn (i.e. Zn exceeds MKM). No sediment concentrations exceeded KM values in pond C and E. This suggest that some elements—particularly Zn—will limit the re-use of the sediments from the older ponds, and they may require costly and laborious clean-up procedures (Zubala et al. 2018) when excavated in the future.

### Priority ranking of ponds and elements

The ponds were then ranked according to their *I*_geo_, RI and RCR values (Table 9). The rankings were similar for all methods except *I*_geo_ was higher in pond E than in pond C. Overall, Pond B was the most contaminated. The degree of contamination was summarised as B >  >  > D ≈ A > C ≈ E. The degree of contamination in ponds C and E was low such that their RCR values matched those of the surrounding soil. This confirmed that the younger ponds (C and E) were less contaminated than the older ponds. In theory, the annual accumulation of trace element should be the same if it is not influenced by other factors. In this case, it was assumed that low rainfall during 2018, 60% less than normal (Swedish Meteorological and Hydrological Institute 2021), contributed to a low accumulation. Additionally, the percentage impervious surface of pond C was low (12%) as the pond was constructed before the industrial area has been fully developed.

**Table 9:** Priority ranking of contamination in the ponds and identified elements of concern based upon *I*_geo_, RI and RCR

Pond	Rank sum-*I*_geo_^1^	Rank RI^2^	Rank sum-RCR^3^	Rank^4^	*I*_geo_^5^	Mean-*Er* > 40^6^	Mean-RCR ≥ 1^7^
	Ranking of ponds				Elements of concern		
**A**	3 (11.8)	3 (593)	3 (11.1)	3	Ag, S, Sb, Zn	Ag, Cd, Sb	Cu, Sb, V, **Zn**
**B**	1 (18.5)	1 (1248)	1 (17.3)	1	**Ag**, Cd, Cu, P, Pb, **S**, **Sb**, **Zn**	Ag, Cd, Sb	Ba, Cd^8^, Co, Cu, Sb, V, **Zn**
**C**	5 (1.0)	4 (134)	4 (6.3)	4	S	None	Co, Ni, V,
**D**	2 (12.6)	2 (646)	2 (12.3)	2	Ag, Cd, Cu, **S**, **Sb**, **Zn**	Ag, Cd, Sb	Co, Cu, Sb, V, **Zn**
**E**	4 (2.1)	5 (117)	5 (3.9)	5	S, Sb	Sb	none
**soil**	n.a.^9^	n.a	5.6	n.a	n.a	n.a	Co, V
List elements of concern					Ag, Cd, Cu, (Pb), Sb, Zn		

^1^Sum *I*_geo_ in parenthesis. When calculating sum *I*_geo_ negative *I*_geo_ values have been set to 0. *I*_geo_ was calculated for all elements except Hg which was below detection limit in all samples. Sum *I*_geo_ has been calculated to enable comparison in the risk ranking of the ponds

^2^Sum Mean Potential Ecological Risk Index in parenthesis. RI was calculated including all elements except Hg, P and S, see also Table 7

^3^Sum Mean RCR in parenthesis. Sum RCR was calculated for all elements except Hg, P and S, see also Table 8

^4^Based upon sum of the 3 ranking methods

^5^Mean *I*_geo_ values classified in Class 2 or higher are included. Values in Class 3 or higher in bold

^6^Mean *Er* values higher than Class 2 are included. Values in bold are Class 3 or higher, see Table 7

^7^Mean RCR > 2 in bold

^8^For one sampling site (B4), the RCR is above 1

^9^n.a. not applicable. Soil is used in calculation of *I*_geo_ and RI

The 3 ranking methods used were originally developed for sediment quality assessment in lakes and watercourses (Müller 1969; Håkanson 1980; Crommentuijn et al. 2000). To our knowledge, this study represented the first use of *I*_geo_ and RI in the risk assessments of stormwater ponds sediment. The RCR-method is rarely used but sediment guidelines are frequently compared to measured values when assessing the quality of stormwater sediment (Färm et al. 2003; Anderson et al. 2004; Färm and Waara 2005; Jang et al. 2010; Blecken et al. 2012; Sharley et al. 2017; Crane 2019). It is encouraging that *I*_geo_, for which there is no need to find neither *Tr* nor PNEC values, provided a similar ranking as the RI and the RCR-method. This suggested that the *I*_geo_ method is suitable for evaluating the risk caused by the accumulation of emerging contaminants in stormwater ponds.

Although the different ranking methods indicated somewhat different elements of concern (see Table 9), their scales for classification were different and P and S were not included in the RI and Sum-RCR values. P and S are not considered toxic trace elements, but they are used in this study to investigate if they are correlated with the accumulated trace elements. Both elements belong to the Group A elements identified in the PCA (see Fig. 5a, b), and they were significantly correlated with the trace elements with a high *I*_geo_ (Fig. 6). P and S are rarely analysed in sediment in stormwater ponds, but the P level measured here was within the same range (358–4130 mg/kg DW) as reported by Istenič et al. 2012 (90–1810 mg/kg DW). The levels for S found in this study (347–3170 mg/kg DW) are within the lower range of the interval found by Blecken et al. in 2012 (358–16,000 mg/kg DW). Finally, while both P and S showed high and significant correlation coefficients with trace elements with a high *I*_geo_, P showed very low accumulation compared to S. This indicated that S was generally a better indicator of contamination than P, but additional studies are necessary to explore if S content can be a general indicator of pollution in stormwater ponds.

It was assumed that the RCR values for Co, Ni and V (> 1) were questionable since *I*_*geo*_ indicated no accumulation of these elements above the background reference soil (Table 9). The NC value used as PNEC was derived for risk assessments made in the Netherlands, which accounted for higher local background concentrations than what we observed in own reference soil (except Co and V; Crommentuijn et al. 2000). There are currently no available environmental quality standard (EQS) for sediment in lakes and watercourses in Sweden for most of the trace elements analysed, except for Cd (EQS = 2.3 mg/kg DW, NC = 1.1 mg/kg DW), Cu (EQS = 36 mg/kg DW, NC = 37 mg/kg DW) and Pb (EQS = 130 mg/kg DW, NC = 132 m/kg DW) (HVMFS2019:25 2019). However, replacing the NCs used in this study with the national EQSs only marginally changed the RCRs for Cu and Pb, although the average RCR for Cd fell under 1. As both the *I*_geo_ and *Er* values indicated that Cd was an element of concern in pond B, Cd was kept as an element of concern overall. Denmark has set a national guideline for V to 23.6 mg/kg DW using the added risk approach (Ministry of Environment and Food of Denmark 2017), which can be added to the background concentration. In our case, the concentration in a sample must have exceeded 71.7 mg/kg DW (i.e. 48.1 + 23.6 = 71.7) to affect the ecosystem negatively, and this level was only exceeded in sample A2 (Supplementary Data Table S2).

The NCs and the national EQSs for Cd, Cu and Pb were calculated under the assumption that the total organic carbon (TOC) was 5%, and that at a higher TOC the trace elements would bind to the organic matter and become biologically unavailable (CIS 2011). Organic C was not measured in this study, but we assume that organic C content would vary depending upon land use and amount of vegetation in the ponds.

Ag is considered an element of concern despite low RCR levels (i.e. RCR < 1) because the *I*_geo_ value indicated extensive accumulation and the *Er* value was high. Cu was also classified as an element of concern even though the RCR was also below 1 because the *I*_geo_ and *Er* value indicated a risk and the KM guideline was exceeded in pond B and D. As and Pb are interesting cases as the *I*_geo_ indicated accumulation in pond A, B and D and the RCRs was below 1, but the KM values for Pb (KM = 50 mg/kg DW; NC = 132 mg/kg DW) and As (KM = 10 mg/kg DW; NC = 31 mg/kg DW) were exceeded in pond B. This was attributed to the setting of the generic guideline values for contaminated soil for both ecological and human risk. The RCR for Ba was above 1 only in pond B, while *I*_geo_ indicated only slight pollution (Class 1) and the *Er* value indicated low risk in the same pond. Consequently, Ba may not be classified as an element of concern. The high RCRs in pond A, B and D for Sb and Zn agreed directly with the *I*_geo_ and *Er* classification and were thereby classed as elements of high concern. Sb and Zn also show high correlation in the PCA (Fig. 5b) and may therefore have a common source of origin. In conclusion, the use and comparison of three risk assessment methods confirmed that both commonly monitored trace elements (Cd, Cu, Zn) and seldom monitored trace elements (Ag, Sb) may cause an ecological risk in the ponds.

For Ag (Färm et al. 2003; Frost et al. 2015; Sharley et al. 2017; Crane 2019) and Sb (Sharley et al. 2017; Crane 2019), we have found only a few records of measurements in stormwater ponds with catchments featuring industrial land, yet the current data showed that monitoring these elements is paramount as they showed very high accumulation compared to the background. Ag is currently used in many nanomaterials in textiles, medical products, food containers, cosmetics, paints and nano-functionalised plastics (McGillicuddy et al. 2017). Meanwhile, Sb is used in catalysts for production of polyethylene terephthalate (PET), and in flame retardants for various materials such as paper, plastic, paints and textiles (Wang et al. 2018); and brake linings (Wang et al. 2018; Müller et al. 2020). It has recently been found in high concentration in plastic waste fractions (Viczek et al. 2020); and shown to be mobilised from microplastic in coastal estuarine sediments when extracted with fluids that simulate the digestive system of sediment dwelling invertebrates (James and Turner 2020). Significantly, Pond D is located near to a municipal recycling plant. As the industrial areas are less than 5 km apart, they may also experience similar sources of local and long-distance transport of airborne pollutants. Clearly, further studies are needed to determine whether the high accumulation of Ag and Sb in the studied stormwater ponds is a local phenomenon or not.

In this study, the total amount of certain elements was measured and risk-assessed. There are several studies that indicate that heavy metals preferentially accumulate in the roots of macrophytes and are therefore unlikely to become bioavailable via plant uptake and translocation (Istenič et al. 2012). Benthic organisms have also been shown to accumulate heavy metals in stormwater ponds such as Cd, Cr, Ni and Cu (Stephansen et al. 2014), but they might not reach toxic levels as was demonstrated by Anderson et al. (2004) and Casey et al. (2006). Recently, Sun et al. (2019) studied the biodiversity in roadside ponds and found that most of the taxa displayed in the ordination diagram were negatively correlated with the pollution levels in the water column and sediments, while a positive correlation with the pond size and the number of neighbouring ponds was observed. Whether the trace elements studied here will cause toxicity and thereby reduce the biodiversity in catchments with industrial activity remains to be explored.

## Conclusions

There is an urgent need to update the list of trace elements that are routinely monitored in sediment in stormwater ponds when evaluating ecological risk, especially in ponds with catchments in areas of industrial land use. The ponds in this study were mainly constructed to protect downstream areas from flooding during high flows and therefore have low hydraulic efficiency. Accordingly, some trace elements that bind to finer particles that did not sediment in the ponds might therefore flow into the river downstream. Currently, there is no information about background concentrations nor the degree of pollution of Ag and Sb in the downstream catchment nor in other catchments in the region of study. Therefore, the development of a program for monitoring numerous trace elements—significantly more than what is currently monitored—is essential. Identifying the sources of the high accumulation of Ag and Sb found in this study is another priority. Finally, we showed that the use and comparison of different assessment methods including multivariate analyses when evaluating ecological risk of stormwater pond sediment are valuable as it highlighted different aspects of risk. For example, information from the geoaccumulation index combined with the multivariate analyses clearly demonstrated that Co, Ni and V were not accumulating, and therefore their risk as identified by the RCR-method was overestimated. On the other hand, for Ag, RCRs are below 1 also in the most contaminated pond B, while the geoaccumulation index and RI indicate high accumulation and high risk, triggering the need for additional studies.

## Supplementary Information

Below is the link to the electronic supplementary material.

Supplementary file1 (DOCX 54 KB)

## Data Availability

All data generated or analysed during this study are included in this published article and its supplementary information files.

## References

[CR1] Aksu A, Yasar D, Uslu O (1998). Assessment of marine pollution in Izmir Bay: heavy metal and organic compound concentrations in surficial sediments. Turk J Eng Environ Sci.

[CR2] Allen D, Haynes H, Arthur S (2017). Contamination of detained sediment in sustainable urban drainage systems. Water.

[CR3] Anderson BC, Bell T, Hodson P, Marsalek J, Watt WE (2004). Accumulation of trace metals in freshwater invertebrates in stormwater management facilities. Water Qual Res Canada.

[CR4] Baekken T (1994). Effects of highway pollutants on a small Norwegian lake. Sci Total Environ.

[CR5] Blecken G-T, Rentz R, Malmgren C, Öhlander B, Viklander M (2012). Stormwater impact on urban waterways in cold climate: variations in sediment metal concentrations due to untreated snowmelt discharge. J Soil Sediments.

[CR6] Blecken G, Al-Rubaei A, Viklander M, Marsalek J (2017) 25 municipal stormwater management ponds in Sweden—survey of the operational status. Bromma: Svenskt Vatten (In Swedish)

[CR7] Casey RE, Simon JA, Atueyi S, Snodgrass JW, Karouna-Renier N, Sparling DW (2006). Temporal trends of trace metals in sediment and invertebrates from stormwater management ponds. Water Air Soil Pollut.

[CR8] CIS (2011) Technical guidance for deriving environmental quality standards, technical report 2011–055. Common implementation strategy for the water framework directive (2000/60/EC)

[CR9] Crane JL (2019). Distribution, toxic potential, and influence of land use on conventional and emerging contaminants in urban stormwater pond sediments. Arch Environ Contam Toxicol.

[CR10] Crommentuijn T, Sijm D, de Bruijn J, van den Hoop M, van Leeuwen K, van de Plassche E (2000). Maximum permissible and negligible concentrations for metals and metalloids in the Netherlands, taking into account background concentrations. J Environ Manag.

[CR11] Duodu GO, Goonetilleke A, Ayoko GA (2016). Comparison of pollution indices for the assessment of heavy metal in Brisbane River sediment. Environ Pollut.

[CR12] ECHA (2016) Guidance on information requirements and chemical safety assessment part E: risk characterisation. Version 3.0. May 2016, ISBN 978–92–9495–055–0

[CR13] Egemose S, Sønderup MJ, Grudinina A, Hansen AS, Flindt MR (2015). Heavy metal composition in stormwater and retention in ponds dependent on pond age, design and catchment type. Environ Technol.

[CR14] European Chemicals Bureau (2003) Technical guidance document on risk assessment. Part II. Environmental risk assessment

[CR15] Frost PC, Song K, Buttle JM, Marsalek J, McDonald A, Xenopoulos MA (2015). Urban biogeochemistry of trace elements: what can the sediments of stormwater tell us?. Urban Ecosyst.

[CR16] Färm C, Waara S (2005). Treatment of stormwater using a detention pond and constructed filters. Urban Water J.

[CR17] Färm C, Johansson D, Kadic Z, Waara S (2003) Characterisation of sediment from storm water tunnels. In: Tiezzi E, Brebbia CA, Usó J-L (eds) Ecosystem and sustainable development, vol 2:1253–1262, WIT Press, Transactions on Ecology and the Environment

[CR18] HVMFS2019:25 (2019) Classification and environmental quality standards for surface water (In Swedish). Swedish Agency for Marine and Water Management

[CR19] Håkanson L (1980). An ecological risk index for aquatic pollution control: a sedimentological approach. Water Res.

[CR20] Istenič D, Arias CA, Vollertsen J, Nielsen AH, Wium-Anderson T, Hvitved-Jacobsen T, Brix H (2012). Improved urban stormwater treatment and pollution removal pathways in amended wet detention ponds. J Environ Sci Health A.

[CR21] James E, Turner A (2020) Mobilisation of antimony from microplastics added to coastal sediment. Environ Pollut 264. 10.1016/j.envpol.2020.114696 10.1016/j.envpol.2020.114696 32388305

[CR22] Jang Y-C, Jain P, Tolaymat T, Dubey B, Singh S, Townsend T (2010). Characterization of roadway stormwater system residuals for reuse and disposal options. Sci Total Environ.

[CR23] Jefferson AJ, Bhaskar AS, Hopkins KG, Fanelli R, Avellaneda PM, McMillan SK (2017). Stormwater management network effectiveness and implications for urban watershed function: a critical review. Hydrol Process.

[CR24] Jiao F, Ren L, Wang X, Liu W (2017). Pollution characteristics and potential ecological risk assessment of metals in the sediments of Xiaoqing River. Jinan Environ Sci Pollut Res.

[CR25] Li W, Lin S, Wang W, Huang Z, Zeng H, Chen X, Zeng F, Fan Z (2020). Assessment of nutrient and heavy metal contamination in surface sediments of the Xiashan stream, eastern Guangdong Province, China. Environ Sci Pollut Res.

[CR26] Maltby L, Forrow DM, Boxall ABA, Calow P, Betton C (1995). The effects of motorway runoff on freshwater ecosystems: 1. Field study. Environ Toxicol Chem.

[CR27] Manoj K, Padhy PK (2014). Distribution, enrichment and ecological risk assessment of six elements in bed sediments of a tropical river, Chottanagpur Plateau: a spatial and temporal appraisal. J Environ Prot.

[CR28] Marsalek J, Urbonas B, Lawrence I, Shilton A (2005). Stormwater management ponds. Pond treatment technology.

[CR29] McGillicuddy E, Murray I, Kavanagh S, Morrison L, Fogarty A, Cormican M, Dockery P, Prendergast M, Rowan N, Morris D (2017). Silver nanoparticles in the environment: sources, detection and ecotoxicology. Sci Total Environ.

[CR30] Ministry of Environment and Food of Denmark. 2017. Bekendtgørelse af lov om vandplanlægning. LBK nr 126 af 26/01/2017. https://www.retsinformation.dk/Forms/R0710.aspx?id=196701

[CR31] Müller G (1969). Index of geoaccumulation in the sediments of the Rhine River. GeoJournal.

[CR32] Müller A, Österlund H, Marsalek J, Viklander M (2020) The pollution conveyed by urban runoff: a review of sources. Sci Total Environ 709. 10.1016/j.scitotenv.2019.136125 10.1016/j.scitotenv.2019.136125 31905584

[CR33] Nika MC, Ntaiou K, Elytis K, Thomaidi VS, Gatidou G, Kalantzi OI, Thomaidis NS, Stasinakis AS (2020) Wide-scope target analysis of emerging contaminants in landfill leachates and risk assessment using Risk Quotient methodology. J Hazard Mater 394. 10.1016/j.jhazmat.2020.122493 10.1016/j.jhazmat.2020.122493 32240898

[CR34] Sharley DJ, Sharp MS, Bourgues S, Pettigrove VJ (2016). Detecting long-term temporal trends in sediment-bound trace metals from urbanised catchments. Environ Pollut.

[CR35] Sharley DJ, Sharp MS, Marshall S, Jeppe M, Pettigrove JV (2017). Linking urban land use to pollutants in constructed wetlands: implications for stormwater and urban planning. Landsc Urban Plan.

[CR36] Stephansen DA, Nielsen AH, Hvitved-Jacobsen T, Arias CA, Brix H, Vollertsen J (2014). Distribution of metals in fauna, flora and sediments of wet detention ponds and natural shallow lakes. Ecol Eng.

[CR37] Sun Z, Sokolova E, Brittain JE, Saltveit SJ, Rauch S, Meland S (2019). Impact of environmental factors on aquatic biodiversity on roadside ponds. Sci Rep.

[CR38] Søberg LC, Vollertsen J, Blecken GT, Haaning Nielsen A, Viklander M (2016). Bioaccumulation of heavy metals in two wet retention ponds. Urban Water J.

[CR39] Swedish Environmental Protection Agency (2009) Guideline values for contaminated soil. Model description and guidelines. (In Swedish) Report 5976

[CR40] Swedish Environmental Protection Agency (2016) Updated list of guideline values (In Swedish). https://www.naturvardsverket.se/upload/stod-i-miljoarbetet/vagledning/fororenade-omraden/berakning-riktvarden/generella-riktvarden-20160707.pdf. Accessed 16 March 2021

[CR41] Swedish Meteorological Institute (SMHI) (2021). https://www.smhi.se/data/meteorologi/kartor/karta/foregaende-ar/nederbord/. Accessed 16 March 2021

[CR42] Viczek SA, Aldrian A, Pomberger R, Sarc R (2020). Origins and carriers of Sb, As, Cd, Cl, Cr Co, Pb, Hg, and Ni in mixed solid waste—a literature-based evaluation. Waste Manag.

[CR43] Wang J-Z, Peng S-C, Chen T-H, Zhang L (2016). Occurrence, source identification and ecological risk evaluation of metal elements in surface sediment: toward a comprehensive understanding of heavy metal pollution in Chaohu Lake, Eastern China. Environ Sci Pollut Res.

[CR44] Wang N, Wang A, Kong L, He M (2018). Calculation and application of Sb toxicity coefficient for potential ecological risk assessment. Sci Total Environ.

[CR45] Wei L, Cai M, Du Y, Tang J, Wu Q, Xiao T, Luo D, Huang X, Liu Y, Fei Y, Chen Y (2019). Spatial attenuation of mining/smelting-derived metal pollution in sediments from tributaries of the Upper Han River, China. Mine Water Environ.

[CR46] Yang J, Wang W, Zhao M, Chen B, Dada OA, Chu Z (2015). Spatial distribution and historical trends of heavy metals in the sediments of petroleum producing regions of the Beibu Gulf, China. Mar Pollut Bull.

[CR47] Zhang H, Jiang Y, Ding M, Xie Z (2017). Level, source identification, and risk analysis of heavy metal in surface sediments from river-lake ecosystems in the Poyang Lake, China. Environ Sci Pollut Res.

[CR48] Zhou S, Di Paolo C, Wu X, Shao Y, Seiler TB, Hollert H (2019). Optimization of screening-level risk assessment and priority selection of emerging pollutants—the case of pharmaceuticals in European surface waters. Environ Int.

[CR49] Zhu X, Ji H, Chen Y, Qiao M, Tang L (2013). Assessment and sources of heavy metals in surface sediments of Miyun Resorvoir, Beijing. Environ Monit Assess.

[CR50] Zubala T, Patro M, Boguta P (2018). Variability of zinc, copper and lead contents in sludge of the municipal stormwater treatment plant. Environ Sci Pollut Res.

