# Supplementary material for: Ecological risk assessment of trace elements accumulated in stormwater ponds within industrial areas
Source: Environ Sci Pollut Res Int. 2021 Dec 21;29(18):27026–41. doi: 10.1007/s11356-021-18102-0 (PMC8989822; doi:10.1007/s11356-021-18102-0)
Supplement: Supplementary file 1 — Supplementary file1 (DOCX 54 KB) [file 11356_2021_18102_MOESM1_ESM.docx]

# Supplementary Data

## Title: Ecological risk assessment of trace elements accumulated in stormwater ponds within industrial areas

## Journal: Environmental Science and Pollution Research

Sylvia Waara^1,2^, Frida Johansson^1^

Department of Environmental and Biosciences, Rydberg Laboratory of Applied Sciences, Halmstad University, Box 823, 301 18 Halmstad

^2^Corresponding address: [sylvia.waara@hh.se](mailto:sylvia.waara@hh.se), phone +46729773608

Supplementary Data Table S1. Generic guideline values for contaminated soil ( Swedish Environmental Protection Agency 2016)

| **Element** | **KM (sensitive land use) mg/kg DW** | **MKM (Less sensitive land use)**  **Mg/kg DW** |
| --- | --- | --- |
| **Antimony** | 12 | 30 |
| **Arsenic** | 10 | 25 |
| **Barium** | 200 | 300 |
| **Cadmium** | 0.8 | 12 |
| **Cobolt** | 15 | 35 |
| **Chromium^1^** | 80 | 150 |
| **Copper** | 80 | 200 |
| **Lead** | 50 | 400 |
| **Mercury** | 0.25 | 2.5 |
| **Nickel** | 40 | 120 |
| **Vanadium** | 100 | 200 |
| **Zinc** | 250 | 500 |

^1^ If Cr (VI) is constituting more than 1 % of the total concentration of Cr also Cr (VI) should be risk assessed.

Supplementary Data Table S2. Trace element concentration, particle size and sediment depth in sediment samples from five stormwater ponds. Values above sensitive land use (KM) in italics, values above less sensitive land use (MKM) in italics and bold. No generic guideline value was available for Ag, P and S.

| **Sample**  **mg/kg DW** | **Ag** | **As** | **Ba** | **Cd** | **Co** | **Cr** | **Cu** | **Ni** | **P** | **Pb** | **S** | **Sb** | **V** | **Zn** | **PSsilt/**  **clay**  **(%)** | **PSsand**  **(%)** | **PSgravel**  **(%)** | **Sediment**  **Depth**  **(cm)** |
| --- | --- | --- | --- | --- | --- | --- | --- | --- | --- | --- | --- | --- | --- | --- | --- | --- | --- | --- |
| **A1:in** | 0.27 | 5.81 | 105 | 0.382 | 9.29 | 32.8 | 42.5 | 22.2 | 1360 | 33.1 | 2460 | 3.68 | 57.5 | *334* | 80.57 | 18.59 | 0.84 | 9.8 |
| **A2:out** | 0.25 | 8.28 | 140 | 0.484 | 11.70 | 45.6 | 52.0 | 29.1 | 1760 | 42.8 | 2140 | 5.50 | 75.3 | *444* | 92.64 | 7.2 | 0.16 | 14.5 |
| **B1:in** | 0.65 | 8.84 | 122 | 0.680 | 9.61 | 30.3 | 60.3 | 22.0 | 3040 | 44.2 | 2290 | 6.41 | 51.4 | ***534*** | 91.38 | 6.4 | 2.22 | 11.0 |
| **B2:in** | 0.55 | *10.40* | 155 | 0.783 | 12.50 | 39.0 | *82.4* | 26.8 | 3730 | *64.2* | 3170 | 8.37 | 67.2 | ***724*** | 86.81 | 5.39 | 7.80 | 20.0 |
| **B3:in** | 0.82 | *10.20* | 135 | 0.779 | 8.13 | 28.6 | *82.5* | 21.1 | 3280 | *65.5* | 1910 | 9.22 | 43.6 | ***818*** | 50.75 | 43.13 | 6.13 | 5.5 |
| **B4:out** | 0.95 | *14.30* | 194 | 1.140 | 9.77 | 36.0 | *121.0* | 32.6 | 4130 | *87.6* | 2860 | *12.30* | 52.2 | ***1190*** | 64.84 | 29.46 | 5.70 | 30.0 |
| **C1:in** | 0.04 | 4.09 | 81 | 0.145 | 13.10 | 37.2 | 21.6 | 34.9 | 693 | 19.5 | 1030 | 0.42 | 53.3 | 92 | 89.08 | 8.78 | 2.15 | 1.0 |
| **C2:out** | 0.03 | 4.00 | 85 | 0.145 | 13.20 | 41.4 | 20.9 | 32.6 | 607 | 18.1 | 845 | 0.33 | 56.6 | 79 | 91.32 | 4.54 | 4.14 | 1.0 |
| **D1:in** | 0.15 | 5.79 | 76 | 0.387 | 8.21 | 19.2 | 62.4 | 16.2 | 1230 | 27.2 | 1160 | 3.98 | 37.2 | *445* | 55.82 | 38.84 | 5.34 | 6.7 |
| **D2:out** | 0.17 | 8.86 | 80 | 0.358 | 8.97 | 22.1 | 55.8 | 17.7 | 1490 | 25.4 | 1040 | 4.09 | 39.2 | *395* | 40.67 | 56.8 | 2.53 | 9.9 |
| **D3:out** | 0.36 | 9.98 | 107 | *0.804* | 11.00 | 28.5 | *126.0* | 20.7 | 1660 | 44.1 | 2370 | 8.49 | 52.7 | ***833*** | 76.08 | 22.03 | 1.88 | 5.0 |
| **E1:in** | 0.04 | 1.47 | 39 | 0.141 | 3.51 | 9.0 | 21.7 | 6.4 | 504 | 7.8 | 1020 | 1.32 | 16.3 | 140 | 16.36 | 80.91 | 2.73 | 8.8 |
| **E2:in** | 0.03 | 0.94 | 46 | 0.050 | 12.80 | 11.3 | 23.5 | 8.6 | 358 | 6.5 | 347 | 0.38 | 15.9 | 92 | 5.06 | 69.12 | 25.82 | 17.5 |
| **E3:out** | 0.03 | 1.74 | 68 | 0.155 | 5.92 | 14.6 | 16.7 | 11.4 | 515 | 11.1 | 782 | 0.77 | 27.7 | 151 | 32.00 | 66.22 | 1.78 | 0.1 |
| **A:soil** | 0.05 | 2.66 | 72 | 0.110 | 9.82 | 27.8 | 17.2 | 21.2 | 653 | 13.3 | 265 | 0.15 | 44.1 | 78 | 89.87 | 10.03 | 0.10 | --- |
| **C:soil** | 0.03 | 5.37 | 91 | 0.192 | *16.10* | 37.5 | 19.0 | 36.5 | 696 | 18.6 | 97 | 0.03 | 49.7 | 72 | 78.75 | 8.72 | 12.53 | --- |
| **E:soil** | 0.02 | 5.01 | 81 | 0.115 | 12.10 | 37.4 | 17.6 | 27.2 | 599 | 14.6 | 57 | 0.18 | 50.6 | 69 | 46.38 | 35.69 | 17.93 | --- |
|  | | | | | | | | | | | | | | | | | | |
| **A:average** | 0.26 | 7.05 | 123 | 0.433 | 10.50 | 39.2 | 47.3 | 25.7 | 1560 | 38.0 | 2300 | 4.59 | 66.4 | *389* | 86.61 | 12.90 | 0.50 | 12.1 |
| **B:average** | 0.74 | *10.94* | 152 | *0.846* | 10.00 | 33.5 | *86.6* | 25.6 | 3545 | *65.4* | 2558 | 9.08 | 53.6 | ***817*** | 73.45 | 21.10 | 5.46 | 16.6 |
| **C:average** | 0.03 | 4.05 | 83 | 0.145 | 13.15 | 39.3 | 21.3 | 33.8 | 650 | 18.8 | 938 | 0.37 | 55.0 | 86 | 90.20 | 6.66 | 3.15 | 1.0 |
| **D:average** | 0.23 | 8.21 | 88 | 0.516 | 9.39 | 23.3 | *81.4* | 18.2 | 1460 | 32.2 | 1523 | 5.52 | 43.0 | ***558*** | 57.52 | 39.22 | 3.25 | 7.2 |
| **E:average** | 0.03 | 1.38 | 51 | 0.115 | 7.41 | 11.6 | 20.6 | 8.8 | 459 | 8.5 | 716 | 0.82 | 20.0 | 128 | 17.81 | 72.08 | 10.11 | 8.8 |
| **Soil:average** | 0.03 | 4.35 | 81 | 0.139 | 12.67 | 34.2 | 17.9 | 28.3 | 649 | 15.5 | 140 | 0.12 | 48.1 | 73 | 71.67 | 18.15 | 10.19 | --- |

Supplementary Data Table S3. Geoaccumulation index. Igeo (Müller 1969) for trace elements measured in sediment in 5 stormwater ponds.

| **sample** | **Igeo-Ag** | **Igeo-As** | **Igeo-Ba** | **Igeo-Cd** | **Igeo-Co** | **Igeo-Cr** | **Igeo-Cu** | **Igeo-Ni** | **Igeo-**  **P** | **Igeo-Pb** | **Igeo-**  **S** | **Igeo-Sb** | **Igeo-**  **V** | **Igeo-Zn** | **Sum Igeo**  **negative values set to 0** |
| --- | --- | --- | --- | --- | --- | --- | --- | --- | --- | --- | --- | --- | --- | --- | --- |
| **A1:in** | 2.56 | -0.17 | -0.22 | 0.87 | -1.03 | -0.65 | 0.66 | -0.94 | 0.48 | 0.51 | 3.55 | 4.35 | -0.33 | 1.61 | 10.56 |
| **A2:out** | 2.40 | 0.34 | 0.20 | 1.21 | -0.70 | -0.17 | 0.95 | -0.54 | 0.85 | 0.88 | 3.35 | 4.93 | 0.06 | 2.02 | 13.00 |
| **B1:in** | 3.81 | 0.44 | 0.00 | 1.71 | -0.98 | -0.76 | 1.16 | -0.95 | 1.64 | 0.93 | 3.45 | 5.15 | -0.49 | 2.29 | 15.48 |
| **B2:in** | 3.56 | 0.67 | 0.34 | 1.91 | -0.60 | -0.40 | 1.62 | -0.66 | 1.94 | 1.47 | 3.92 | 5.54 | -0.10 | 2.73 | 17.83 |
| **B3:in** | 4.15 | 0.65 | 0.15 | 1.90 | -1.23 | -0.84 | 1.62 | -1.01 | 1.75 | 1.49 | 3.19 | 5.67 | -0.73 | 2.90 | 18.53 |
| **B4:out** | 4.36 | 1.13 | 0.67 | 2.45 | -0.96 | -0.51 | 2.17 | -0.38 | 2.08 | 1.91 | 3.77 | 6.09 | -0.47 | 3.44 | 22.23 |
| **C1:in** | -0.41 | -0.67 | -0.60 | -0.52 | -0.54 | -0.47 | -0.32 | -0.28 | -0.49 | -0.25 | 2.30 | 1.23 | -0.44 | -0.24 | 1.23 |
| **C2:out** | -0.63 | -0.70 | -0.52 | -0.52 | -0.53 | -0.31 | -0.36 | -0.38 | -0.68 | -0.36 | 2.01 | 0.85 | -0.35 | -0.48 | 0.85 |
| **D1:in** | 1.70 | -0.17 | -0.68 | 0.89 | -1.21 | -1.42 | 1.21 | -1.39 | 0.34 | 0.23 | 2.47 | 4.46 | -0.96 | 2.02 | 10.52 |
| **D2:out** | 1.87 | 0.44 | -0.62 | 0.78 | -1.08 | -1.22 | 1.05 | -1.26 | 0.61 | 0.13 | 2.31 | 4.50 | -0.88 | 1.85 | 10.18 |
| **D3:out** | 2.97 | 0.61 | -0.19 | 1.95 | -0.79 | -0.85 | 2.23 | -1.04 | 0.77 | 0.92 | 3.50 | 5.56 | -0.45 | 2.93 | 17.16 |
| **E1:in** | -0.41 | -2.15 | -1.66 | -0.56 | -2.44 | -2.52 | -0.31 | -2.74 | -0.95 | -1.58 | 2.28 | 2.87 | -2.15 | 0.36 | 3.23 |
| **E2:in** | -0.63 | -2.80 | -1.41 | -2.06 | -0.57 | -2.18 | -0.19 | -2.31 | -1.44 | -1.83 | 0.73 | 1.07 | -2.18 | -0.25 | 1.07 |
| **E3:out** | -0.89 | -1.91 | -0.85 | -0.43 | -1.68 | -1.81 | -0.69 | -1.90 | -0.92 | -1.07 | 1.90 | 2.08 | -1.38 | 0.46 | 2.08 |

Supplementary Data Table S4. Risk factor (*Er*) and Potential Risk Index (RI) for trace elements in sediment in five stormwater ponds. The Potential Risk Index was developed by Håkanson (1980) and the Tr values used in this study are presented in Table 5 in the article.

| **sample**  **(Tr value)** | **Er-**  **Ag (17.5)** | **Er-**  **As (10)** | **Er-**  **Ba (2)** | **Er-**  **Cd (30)** | **Er-**  **Co (5)** | **Er-**  **Cr (2)** | **Er-**  **Cu (5)** | **Er-**  **Ni (5)** | **Er-**  **Pb (5)** | **Er-**  **Sb (7)** | **Er-**  **V (2)** | **Er-**  **Zn (1)** | **RI (sum Er values)** | **% Ag of RI** | **% Cd of RI** | **% Sb of RI** | **% Ag+Cd+Sb**  **of RI** |
| --- | --- | --- | --- | --- | --- | --- | --- | --- | --- | --- | --- | --- | --- | --- | --- | --- | --- |
| **A1:in** | 154.6 | 13.37 | 2.58 | 82.45 | 3.67 | 20.29 | 11.85 | 3.92 | 10.68 | 214 | 2.39 | 4.58 | 524 | 29 | 16 | 41 | 86 |
| **A2:out** | 138.8 | 19.05 | 3.44 | 104.46 | 4.62 | 28.21 | 14.50 | 5.14 | 13.81 | 320 | 3.13 | 6.08 | 661 | 21 | 16 | 48 | 85 |
| **B1:in** | 368.1 | 20.34 | 3.00 | 146.76 | 3.79 | 18.74 | 16.81 | 3.89 | 14.26 | 373 | 2.14 | 7.32 | 978 | 38 | 15 | 38 | 91 |
| **B2:in** | 309.2 | 23.93 | 3.81 | 168.99 | 4.93 | 24.12 | 22.97 | 4.73 | 20.71 | 487 | 2.79 | 9.92 | 1083 | 29 | 16 | 45 | 89 |
| **B3:in** | 466.1 | 23.47 | 3.32 | 168.13 | 3.21 | 17.69 | 23.00 | 3.73 | 21.13 | 536 | 1.81 | 11.21 | 1279 | 36 | 13 | 42 | 92 |
| **B4:out** | 539.2 | 32.90 | 4.77 | 246.04 | 3.85 | 22.27 | 33.74 | 5.76 | 28.26 | 716 | 2.17 | 16.31 | 1651 | 33 | 15 | 43 | 91 |
| **C1:in** | 19.8 | 9.41 | 1.99 | 31.29 | 5.17 | 23.01 | 6.02 | 6.17 | 6.29 | 25 | 2.21 | 1.27 | 137 | 14 | 23 | 18 | 55 |
| **C2:out** | 17.0 | 9.20 | 2.10 | 31.29 | 5.21 | 25.61 | 5.83 | 5.76 | 5.84 | 19 | 2.35 | 1.08 | 130 | 13 | 24 | 15 | 52 |
| **D1:in** | 85.5 | 13.32 | 1.87 | 83.53 | 3.24 | 11.88 | 17.40 | 2.86 | 8.77 | 232 | 1.55 | 6.10 | 468 | 18 | 18 | 50 | 86 |
| **D2:out** | 95.7 | 20.38 | 1.96 | 77.27 | 3.54 | 13.67 | 15.56 | 3.13 | 8.19 | 238 | 1.63 | 5.41 | 484 | 20 | 16 | 49 | 85 |
| **D3:out** | 205.0 | 22.96 | 2.63 | 173.53 | 4.34 | 17.63 | 35.13 | 3.66 | 14.23 | 494 | 2.19 | 11.42 | 987 | 21 | 18 | 50 | 88 |
| **E1:in** | 19.8 | 3.38 | 0.95 | 30.43 | 1.38 | 5.54 | 6.05 | 1.13 | 2.51 | 77 | 0.68 | 1.92 | 151 | 13 | 20 | 51 | 84 |
| **E2:in** | 17.0 | 2.15 | 1.13 | 10.79 | 5.05 | 6.99 | 6.55 | 1.52 | 2.11 | 22 | 0.66 | 1.26 | 77 | 22 | 14 | 29 | 65 |
| **E3:out** | 14.2 | 4.00 | 1.67 | 33.45 | 2.34 | 9.03 | 4.66 | 2.01 | 3.58 | 45 | 1.15 | 2.07 | 123 | 12 | 27 | 36 | 75 |

Supplementary Data Table S5.Risk Characterisation Ratios (RCRs) for sediment samples from five stormwater ponds. Reference soil was sampled around the ponds. The PNEC values used are the Negligible Concentrations (in parenthesis) for sediment derived by Crommentuijn et al. (2000).

| **Sample**  **(PNEC)** | **RCR-Ag**  **(1.5)** | **RCR-As**  **(31)** | **RCR-Ba**  **(157)** | **RCR-Cd**  **(1.1)** | **RCR-Co**  **(9.1)** | **RCR-Cr**  **(116)** | **RCR-Cu**  **(36)** | **RCR-Ni**  **(35)** | **RCR-Pb**  **(132)** | **RCR-Sb**  **(3.2)** | **RCR-V**  **(42)** | **RCR-Zn**  **(145)** | **sum-RCR** |
| --- | --- | --- | --- | --- | --- | --- | --- | --- | --- | --- | --- | --- | --- |
| **A1:in** | 0.18 | 0.19 | 0.67 | 0.35 | 1.02 | 0.28 | 1.18 | 0.63 | 0.25 | 1.15 | 1.37 | 2.30 | 9.58 |
| **A2:out** | 0.16 | 0.27 | 0.89 | 0.44 | 1.29 | 0.39 | 1.44 | 0.83 | 0.32 | 1.72 | 1.79 | 3.06 | 12.61 |
| **B1:in** | 0.43 | 0.29 | 0.78 | 0.62 | 1.06 | 0.26 | 1.68 | 0.63 | 0.33 | 2.00 | 1.22 | 3.68 | 12.98 |
| **B2:in** | 0.36 | 0.34 | 0.99 | 0.71 | 1.37 | 0.34 | 2.29 | 0.77 | 0.49 | 2.62 | 1.60 | 4.99 | 16.86 |
| **B3:in** | 0.55 | 0.33 | 0.86 | 0.71 | 0.89 | 0.25 | 2.29 | 0.60 | 0.50 | 2.88 | 1.04 | 5.64 | 16.54 |
| **B4:out** | 0.63 | 0.46 | 1.24 | 1.04 | 1.07 | 0.31 | 3.36 | 0.93 | 0.66 | 3.84 | 1.24 | 8.21 | 23.00 |
| **C1:in** | 0.02 | 0.13 | 0.51 | 0.13 | 1.44 | 0.32 | 0.60 | 1.00 | 0.15 | 0.13 | 1.27 | 0.64 | 6.35 |
| **C2:out** | 0.02 | 0.13 | 0.54 | 0.13 | 1.45 | 0.36 | 0.58 | 0.93 | 0.14 | 0.10 | 1.35 | 0.54 | 6.27 |
| **D1:in** | 0.10 | 0.19 | 0.49 | 0.35 | 0.90 | 0.17 | 1.73 | 0.46 | 0.21 | 1.24 | 0.89 | 3.07 | 9.79 |
| **D2:out** | 0.11 | 0.29 | 0.51 | 0.33 | 0.99 | 0.19 | 1.55 | 0.51 | 0.19 | 1.28 | 0.93 | 2.72 | 9.59 |
| **D3:out** | 0.24 | 0.32 | 0.68 | 0.73 | 1.21 | 0.25 | 3.50 | 0.59 | 0.33 | 2.65 | 1.25 | 5.74 | 17.51 |
| **E1:in** | 0.02 | 0.05 | 0.25 | 0.13 | 0.39 | 0.08 | 0.60 | 0.18 | 0.06 | 0.41 | 0.39 | 0.97 | 3.52 |
| **E2:in** | 0.02 | 0.03 | 0.29 | 0.05 | 1.41 | 0.10 | 0.65 | 0.25 | 0.05 | 0.12 | 0.38 | 0.64 | 3.97 |
| **E3:out** | 0.02 | 0.06 | 0.43 | 0.14 | 0.65 | 0.13 | 0.46 | 0.33 | 0.08 | 0.24 | 0.66 | 1.04 | 4.24 |
| **A:soil** | 0.03 | 0.09 | 0.46 | 0.10 | 1.08 | 0.24 | 0.48 | 0.61 | 0.10 | 0.05 | 1.05 | 0.54 | 4.81 |
| **C:soil** | 0.02 | 0.17 | 0.58 | 0.17 | 1.77 | 0.32 | 0.53 | 1.04 | 0.14 | 0.01 | 1.18 | 0.50 | 6.44 |
| **E:soil** | 0.01 | 0.16 | 0.52 | 0.10 | 1.33 | 0.32 | 0.49 | 0.78 | 0.11 | 0.06 | 1.20 | 0.47 | 5.56 |

**References**

Crommentuijn T, Sijm D, de Bruijn J, van den Hoop M, van Leeuwen K, van de Plassche, E (2000) Maximum permissible and negligible concentrations for metals and metalloids in the Netherlands, taking into account background concentrations. J Environ Manage 60:121-143.

Håkanson L (1980) An ecological risk index for aquatic pollution control: a sedimentological approach. Water Res 14:975-1001.

Müller G (1969) Index of geoaccumulation in the sediments of the Rhine River. Geojournal 2:108-118.

Swedish Environmental Protection Agency (2016). Updated list of guideline values. (In Swedish) <https://www.naturvardsverket.se/upload/stod-i-miljoarbetet/vagledning/fororenade-omraden/berakning-riktvarden/generella-riktvarden-20160707.pdf>
